# microRNA‐143‐3p attenuated development of hepatic fibrosis in autoimmune hepatitis through regulation of TAK1 phosphorylation

**DOI:** 10.1111/jcmm.14750

**Published:** 2019-12-06

**Authors:** Hanxiao Tu, Dazhi Chen, Chao Cai, Qianjing Du, Hongwei Lin, Tongtong Pan, Lina Sheng, Yuedong Xu, Teng Teng, Jingjing Tu, Zhuo Lin, Xiaodong Wang, Rui Wang, Lanman Xu, Yongping Chen

**Affiliations:** ^1^ Department of Infectious Diseases Wenzhou Key Laboratory of Hepatology The First Affiliated Hospital of Wenzhou Medical University Hepatology Institute of Wenzhou Medical University Wenzhou China; ^2^ Department of Gastroenterology The First Hospital of Peking University BeiJing China; ^3^ Department of Infectious Diseases The Affiliated Yiwu Central Hospital of Wenzhou Medical University Yiwu China; ^4^ Department of Infectious Diseases and Liver Diseases Ningbo Medical Center Lihuili Hospital Ningbo China; ^5^ Department of Infectious Diseases and Liver Diseases The Affiliated Lihuili Hospital of Ningbo University Ningbo China

**Keywords:** autoimmune hepatitis, gene therapy, liver fibrosis, miR‐143, TAK1

## Abstract

Autoimmune hepatitis (AIH) is a chronic liver disease due to autoimmune system attacks hepatocytes and causes inflammation and fibrosis. Intracellular signalling and miRNA may play an important role in regulation of liver injury. This study aimed to investigate the potential roles of microRNA 143 in a murine AIH model and a hepatocyte injury model. Murine AIH model was induced by hepatic antigen S100, and hepatocyte injury model was induced by LPS. Mice and AML12 cells were separated into six groups with or without the treatment of miRNA‐143. Inflammation and fibrosis as well as gene expression were examined by different cellular and molecular techniques. The model was successfully established with the elevation of ALT and AST as well as inflammatory and fibrotic markers. Infection or transfection of mir‐143 in mice or hepatocytes significantly attenuated the development of alleviation of hepatocyte injury. Moreover, the study demonstrated phosphorylation of TAK1‐mediated miRNA‐143 regulation of hepatic inflammation and fibrosis as well as hepatocyte injury. Our studies demonstrated a significant role of miRNA‐143 in attenuation of liver injury in AIH mice and hepatocytes. miRNA‐143 regulates inflammation and fibrosis through its regulation of TAK1 phosphorylation, which warrants TAK1 as a target for the development of new therapeutic strategy of autoimmune hepatitis.

## 1 INTRODUCTION

Autoimmune hepatitis (AIH) is a chronic disease of the liver due to body's immune system attacks liver cells and causes series consequences in the liver such as inflammation, fibrosis and even liver failure.^1^ Approximately one‐third of patients with AIH have evidence of cirrhosis at the time of initial diagnosis.^2^, ^3^ However, patients with AIH progress to cirrhosis at variable rates (ranging from 0.1% to 8.1% annually) despite treatment with immunosuppressive therapies.^4^, ^5^ Moreover, patients with AIH respond well to immunosuppressive treatment if started promptly. If patients left untreated, symptomatic AIH can progress to liver failure, which requires liver transplantation.^3^

In recent years, microRNAs (miRNAs) have been recognized as one of the most important regulators in post‐transcriptional regulation of gene expression. The miRNAs are a class of approximately 22 nucleotide‐non‐coding RNAs, which function as RNA interfering to cause mRNA degradation or translational repression.^6^ Role of miRNAs has been shown in different diseases such as fibrosis, for example miRNA‐143 involving in the progression of human cardiac fibrosis after myocardial infarction,^7^ inhibition of hyperplastic scar formation 8 and regulation of collagen type III expression in stromal fibroblasts of scirrhous‐type gastric cancer.^9^ Moreover, microRNA‐143 also plays an important role in infectious liver disease like hepatitis B,^10^, ^11^ hepatitis C^12^ and/or *Schistosoma japonicum*.^13^ Although the mechanisms underlying these effects are not fully known, several studies have shown involvement of sprouty3 (SPRY3)^7^ and Akt/mTOR pathway^8^ in miRNA regulation of gene expression.

Transforming growth factor β‐activated kinase 1 (TAK1 or MAP3K7) has documented as one of the potential targets of microRNA‐143. TAK1 and its binding partners (TAB1, TAB2 or TAB3) are key signalling components of nuclear factor‐κB (NF‐κB) and mitogen‐activated protein kinase (MAPK) signalling pathways.^14^ TAK1 is involved in the regulation of inflammation through its phosphorylation and activation of the IkB kinase (IKK)‐NF‐κB complex and MAPK kinase.^15^, ^16^, ^17^ However, whether microRNA‐143 mitigates liver inflammation and fibrosis by targeting the TAK1 in AIH is still not known. Therefore, the current study examines the effect of microRNA‐143 on inflammation and fibrosis in an murine model of autoimmune hepatitis. Murine hepatocytes—AML12 cell line are also challenged with LPS to examine protective effects of microRNA‐143 on cells.

## 2 MATERIALS AND METHODS

### 2.1 Materials

LPS (L6386) was purchased from Sigma. Antibodies against TAK1 (5206), P‐TAK1 (9339), NF‐κB (8242S), iκB‐α (4812S) and GADPH (5174) were purchased from Cell Signaling Technology, and COL‐4 (ab6586), TGF‐β (ab92486), CTGF, lamin B (ab133741) and α‐SMA (ab5694) were purchased from Abcam. Mouse CHI3L1 assay Kit was a gift from Hangzhou Proprium Biotech Company Limited. Complete Freund's adjuvant was purchased from Solarbio (Solarbio). Mouse TNF‐α ELISA kit was purchased from the eBioscience (eBioscience).

Establishment of murine experimental model of autoimmune hepatitis with hepatic S100 injection.

All animal care and experimental procedures were approved by the Wenzhou Medical University Animal Policy and Welfare Committee (Approval Document No. wydw2017‐0023), and all animals received humane care according to the National Institutes of Health guidelines (Guide for the care and use of laboratory animals). Male C57BL/6 mice, weighing 23‐25 g, were maintained under specific pathogen‐free conditions with a 12:12‐hour light‐dark cycle at a constant room temperature and fed with standard rodent diet. The animals were acclimatized to the laboratory for at least 2 weeks before initiation of the studies.

For hepatic S100 preparation, ten mice were killed using sodium pentobarbital anaesthesia and liver antigens S100 were prepared freshly after perfusion of livers with phosphate‐buffered saline (PBS) as previously described.^18^ Briefly, the liver was minced and homogenized with cold PBS on ice and subsequently centrifuged at 150 g for 10 min. The supernatants were further centrifuged at 100 000 g for 1 hour, and resulting supernatants were called S100,^18^ which was used for further separation by concentrating to 5 mL using an Amicon Ultra‐15 filter (Millipore, USA) and then passing through a 90 cm CL‐6B Sepharose column (Pharmacia) with the AKTA pure (GE Healthcare). There were three protein peaks (Figure S1B) collected from the column with the peak 2 was toxic components and the peak 1 and peak 3 were safe components as liver antigen. In this experiment, the peak 1 (small molecular fraction) components with the concentration of 0.5‐2.0 g/L were used. Moreover, for immunization, the liver S100 antigen was emulsified in an equal volume of complete Freund's adjuvant (Solarbio). Mice were injected intraperitoneally with this mixture on day 1 and a repeat injection on day 7. To evaluate the disease severity, three mice were killed for histology and blood biochemistry assay. During the experiment, five mice died. Four weeks after the administration, mice were terminated under anaesthesia by 100 mg/kg bodyweight ketamine hydrochloride (Ketanest, Pfizer) and 16 mg/kg bodyweight xylazine hydrochloride (Rompun 2%, Bayer, Leverkusen). Blood samples were collected and centrifuged at 1500 g, 4°C for 10 minutes to collect the serum. Liver tissues were embedded in 4% paraformaldehyde or snap‐frozen in liquid nitrogen for further analysis.

### 2.2 Treatment of miRNA‐143 in murine model of autoimmune hepatitis

The miRNA‐143 was constructed into Adeno‐associated virus (AAV) by the HANBIO. Three AAV constructs of AAV8‐microRNA143‐GFP, AAV8‐microRNA143‐spong off‐GFP and AAV8‐negative control‐GFP were purchased from the HANBIO. These AAV8 viruses were maintained and purified according to the standard protocol provided by the company.^19^

**Figure 1:**
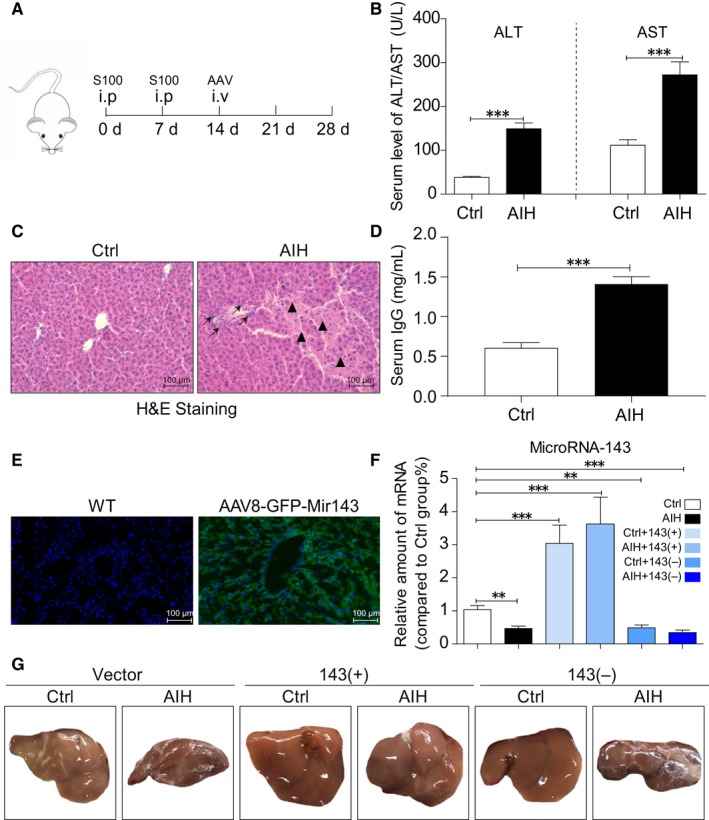
Establishment of mice AIH model and transfection of AAV‐Micro RNA 143. A, The time‐point of autoimmune hepatitis mice establishment and AAV injection. B, The serum levels of ALT and AST in control and AIH group. C, Representative H&E staining of liver tissues showing the structural deficits between the Ctrl and AIH group. Black arrow highlights the lymphocytic infiltration, and black triangle highlights the hepatocyte necrosis. D, IgG levels in mouse serum in Ctrl and AIH groups determined by ELISA. E, GFP immunostaining of liver tissues (shown in green). Slides were counterstained with DAPI. F, mRNA levels of microRNA‐143 in the liver tissues (G) The liver morphology of each group. (*P < .05, **P < .01, ***P < .001, ns = no significance, [n = 7‐9])

Sixty mice were divided into control and AIH groups with each group containing thirty mice. Each group was further divided into three groups (ten mice in each group) of AAV8‐negative control‐GFP (vector), AAV8‐microRNA143‐GFP (143+) and AAV8‐microRNA143‐spong off‐GFP (143‐) in random. Each mouse was injected with 1.0 × 10^11^ copies of AAV8 viruses. The protocol of administration of hepatic antigen S100 and AAVs was outlined in Figure 1.

### 2.3 Liver histopathology examination

Four weeks after S100 first administration, liver tissues were collected from control mice and AIH mice, and then fixed in 4% paraformaldehyde. Tissues were then embedded in paraffin, and 5‐μm sections were prepared. The sections were then routinely stained with H&E, Sirius red and Masson to evaluate the lymphocytic infiltration, hepatocyte necrosis and fibrosis content. Specimens were observed under a light microscope (Nikon).

For immune histochemical staining, sections were deparaffinized and rehydration. Sections were treated with 3% H2O2 for 30 minutes to block endogenous peroxidase activity and then with 1% BSA in PBS for 30 minutes. Slides were incubated with primary antibody anti‐TNF‐a antibody (1:200) overnight at 4°C. PE‐conjugated secondary antibody (anti‐rabbit IgG, at 1:200) was used for detection TNF‐a. Slides were counterstained haematoxylin for 5 minutes, dehydrated and mounted. Images were viewed by a bright field microscope (Nikon).

### 2.4 Cell culture and reagents

AML12 cells are a cell line established from hepatocytes of male mouse, which is kindly provided by the Stem Cell Bank, Chinese Academy of Sciences (Chinese Academy of Sciences, CSCP‐550). AML12 cells were cultured in DMEM/F12 (Gibco) medium supplemented with 10% foetal bovine serum (Gibco), 40 ng/mL dexamethasone, 1% ITS (Sigma) and 1% penicillin‐streptomycin mixture at 37°C in a humidified 5% CO2 incubator.

For in vitro experiment, AML12 cells were plated in 6‐well plates at concentration of 1 × 10^5^ cells. Cells were maintained at 37°C in a humidified atmosphere containing 5% CO2. After cells grown to 80% confluence, cells were transfected with miRNAs of negative control, miRNA‐143 (sequence of 5′‐UGAGAUGAAGCACUGUAGCUC −3′) and miRNA‐143 inhibitors (sequence of 5′‐UUUGUACUACACAAAAGUACUG −3′), respectively, with LipofectAMINE™ 2000 (Thermo Fisher) according to the manufacturer's procedure. After transfection, supernatants were discarded and cells were further cultured with OPTI medium (Gibco) for 6 hours and then in DMEM/F12 for 24 hours.

### 2.5 RNA isolation and qRT‐PCR analysis

Total RNA was isolated from liver tissue or culture cells after infection or transfection of control, miRNA‐143 and miRNA inhibitors by TRIzol reagent (Life Technologies) according to the manufacturer's protocol. Total microRNA was extracted from liver tissue or culture cells using a miRcute miRNA isolation kit (TIANGEN BIOTECH (BEIJING) as described by the manufacturer. Subsequently, total RNA was reverse‐transcribed to cDNA using a RevertAid First Strand cDNA Kit (Thermo Fisher). The products were subjected to real‐time PCR analysis using a CFX96™ Real‐time System (Bio‐Rad) with a Power Sybr Green PCR Master Mix (Bio‐Rad). Primers for gene amplification were synthesized and obtained from GENTEC. Sequences of primers are presented in Table S1. The mRNA data were normalized to β‐actin housekeeping gene. RT‐qPCR assay was performed in duplicate.

### 2.6 NF‐κB staining

Seed AML12 cells were cultured in a 10‐mm cell dish. After treatment, cells were fixed with 4% paraformaldehyde, permeabilized with 0.1% Triton X‐100 and stained with anti NF‐κB antibody (1:200)  for fixation overnight at 4°C. PE‐conjugated secondary antibody (1:200) was used and followed. Nuclei were stained with the DAPI at room temperature. Fluorescent images were viewed and captured by fluorescence microscope (Nikon). Images were captured and quantified by ImageJ (https://imagej.nih.gov/ij/).

### 2.7 Western blot analysis

Total proteins from liver tissue (30‐50 mg) and cells were isolated using lysis buffer (AR0101/0103, Boster Biological Technology Co. Ltd) supplemented with a protease inhibitor—phenylmethanesulfonyl fluoride (PMSF, Beyotime) and quantified by the BCA protein assay kit (Beyotime). After heat denaturation at 100℃ for 10 minutes, fifty microgram of liver tissue and cell lysates were separated by 10% sodium dodecyl sulphate‐polyacrylamide gel electrophoresis and transferred to polyvinylidene fluoride membranes. The membranes were incubated with the following primary antibodies (TAK1, P‐TAK1, NF‐κB, iκB‐α, lamin B and GADPH from Cell Signaling Technology and COL‐4, TGF‐β, CTGF and α‐SMA from Abcam) at 4°C overnight followed by secondary antibodies conjugated with horseradish peroxidase at room temperature for 1 hour. The bands were visualized by using the enhanced chemiluminescence reagent (Bio‐Rad). The density of protein bands was analysed using Image J analysis software version 1.38e and normalized to their respective control.

### 2.8 Examination of serum enzyme and cytokine levels

The serum was isolated from blood samples of each group by 251.55 *g* centrifugation for 10 minutes. According to the manufacturer's protocol, the serum levels of alanine transaminase (ALT) and aspartate transaminase (AST) were evaluated using an automatic biochemistry analyzer (Abbott Laboratories). Scrum TNF‐α levels were measured using mice ELISA kit (eBioscience). Scrum CHI3L1 levels were measured using mice CHI3L1 assay Kit (Hangzhou Proprium Biotech Company Ltd.). Scrum IgG levels were measured using mice ELISA kit (70‐EK271‐96, Multi Science (LIANKE) Biotech, Co. LTD). All experiments were according to the manufacturers’ instructions.

### 2.9 Statistical analysis

All experiments are randomized and blinded. Data represented three independent experiments for cell culture and mice (n = 7 to 9) for in vivo experiment. Data were expressed as means ± SEM. The exact group size (n) for each experimental group/condition is provided, and ‘n’ refers to independent values, not replicates. Statistical analysis was performed with GraphPad Prism 8.0 software. We used one‐way ANOVA followed by Dunnett's post hoc test when comparing more than two groups of data and one‐way ANOVA, non‐parametric Kruskal‐Wallis test, followed by Dunn's post hoc test when comparing multiple independent groups. *P* values of ˂.05 were considered to be statistically significant. Post‐tests were run only if *F* achieved *P* < .05, and there was no significant variance in homogeneity.

## 3 RESULTS

### 3.1 Result 1: Establishment of murine AIH model and transfection of AAV‐Micro RNA 143

As the previous reported,^20^ we first established the mice model of autoimmune hepatitis. The schedule to induce autoimmune hepatitis is shown in Figure 1A. Injection of S100 antigen was performed on day 0 and day 7. The administration of recombinant AAVs was performed on day 14 in tail vein injection. Mice were killed at the end of experiment on day 28. As shown in Figure 1B and D, a significant elevation of serum transaminase (ALT and AST) and immunoglobulin G level in AIH mice indicated success of establishment of murine model of autoimmune hepatitis. Besides, the H&E staining (Figure 1C) also confirms the above conclusions with the evidence of structural alterations in S100‐challenged mice liver including lymphocytic infiltration (black arrow) and hepatocyte necrosis (black triangle).

Expression of miRNA‐143 is shown in Figure 1E and F. There is clear presentation of miRNA‐143 in the liver when AAV‐miRNA‐143 was infected. Then, we investigated which site of microRNA 143 plays the more important role in S100‐stimulated AIH mice model. We measured the different levels of miR‐143‐3P and miR‐143‐5P in liver. As shown in SF1C, the content of miR‐143‐3P in the liver of mice was significantly higher than 5p, suggesting that miR‐143‐3P is the main site.

Moreover, the sizes of the liver in six different groups were presented in Figure 1G. There is a dramatic decrease in liver sizes in AIH group compared with that in control group except the mice treated with miRNA‐143 in AIH group. There is no dramatic difference in liver size between control and AIH when miRNA‐143 was administrated. This finding suggests a significant role of miRNA‐143 in AIH.

### 3.2 Result 2: MicroRNA‐143 mediates liver function and inflammation in mice AIH model

**Figure 2:**
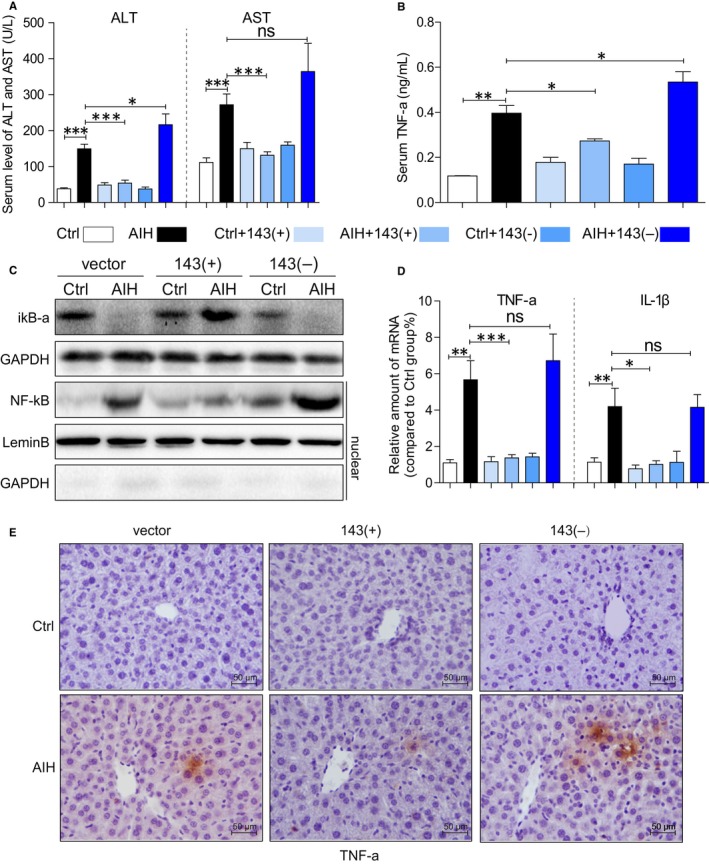
MicroRNA‐143 mediates liver function and inflammation in mice AIH model. A, The serum levels of ALT and AST in each group. B, The serum levels of TNF‐a in each group. C, Representative Western blot analysis of iκB‐a and NF‐κB (nuclear) in liver tissues. GAPDH and lamin B used as loading control. D, mRNA levels of TNF‐a and IL‐1β in the liver tissues. E, Representative TNF‐a immunohistochemistry staining of liver tissues showing effect of microRNA‐143 on S100‐induced liver inflammation. (*P < .05, **P < .01, ***P < .001, ns = no significance [n = 7‐9])

Inspired by the different change in liver morphology in each group, we proposed an assumption that the overexpression of miR‐143 may prohibit the development of AIH. Further investigation of liver function in different treatment groups was shown in Figure 2A. There were significant increases in ALT and AST in AIH mice except the mice were treated with miRNA‐143, and however, overexpression of miR‐143 could prevent it meanwhile.

Inflammatory cytokine and intracellular signalling molecules were then examined as shown in Figure 2. There is a significant increase in TNF‐a level at serum (Figure 2B) and the liver tissue (Figure 2D and E) in AIH mice, and miRNA‐143 treatment attenuated the AIH‐induced liver inflammation. Moreover, intracellular signing molecules are also altered. In parallel to these findings, the degradation of iκB‐α and the nuclear transfection of NF‐κB was obviously increased in AIH mice (Figure 2C). These changes are prevented in mice treated with miR‐143, and no alteration happens in mice treated with down‐regulation of miR‐143. These results demonstrated that miR‐143 prevents S100‐induced liver inflammation and preserves liver function.

### 3.3 Result 3: MicroRNA‐143 mediates liver fibrosis in AIH mice

Since inflammation and fibrosis are two important pathophysiological processes and our data showed the liver appeared to be smaller in AIH groups except the group with miRNA‐143 treatment, it suggests that there might be fibrosis or cirrhosis in the liver.

**Figure 3:**
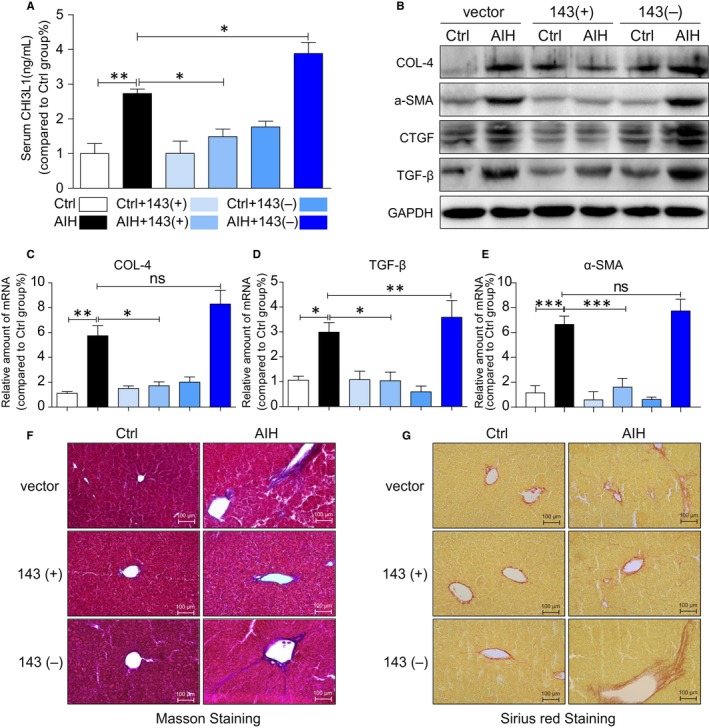
MicroRNA‐143 mediates liver fibrosis in AIH mice. A, The serum levels of CH3L1 in each group. B, Representative Western blot analysis of Col‐4, a‐SMA, CTGF and TGF‐ β in liver tissues. GAPDH used as loading control. C‐E, mRNA levels of CoL‐4 (C), TGF‐β (D) and a‐SMA (E) in liver tissues. F‐G, Fibrosis and connective tissue deposition in liver tissues of S100‐challenged mice. F, shows representative micrographs of Masson trichrome staining, and (G) shows representative micrograph of Sirius Red staining. (*P < .05, **P < .01, ***P < .001, ns = no significance [n = 7‐9])

Therefore, fibrosis parameters were investigated in the blood and the liver. As shown in Figure 3A, serum marker CHI3L1 was significantly elevated in AIH mice compared with control mice except mice treated with miRNA‐143. CHI3L1 is liver‐enriched and non‐invasive biomarker used to stage and diagnoses substantial hepatic fibrosis.^21^ Together, the protein of COL‐4, α‐SMA, CTGF and TGF‐β (Figure 3B) and mRNA levels of Col‐4 (Figure 3C), TGF‐β (Figure 3D) and α‐SMA (Figure 3E) were all increased in liver tissues of AIH mice except mice treated with miRNA‐143. Moreover, miRNA‐143 attenuated the development of liver fibrosis in mice treated with miRNA‐143 as shown in Masson's trichrome (Figure 3F) and Sirius Red staining (Figure 3F) of liver tissues.

### 3.4 Result 4: Identification of TAK1 as a direct MicroRNA‐143 target

In order to find out what is the poential mechanism of miRNA‐143 in attenuation of liver inflammation and fibrosis, the microRNA database (miRbase website) was used to predict the genes regulated by miRNA‐143, where the genes associated with inflammation were selected.

**Figure 4:**
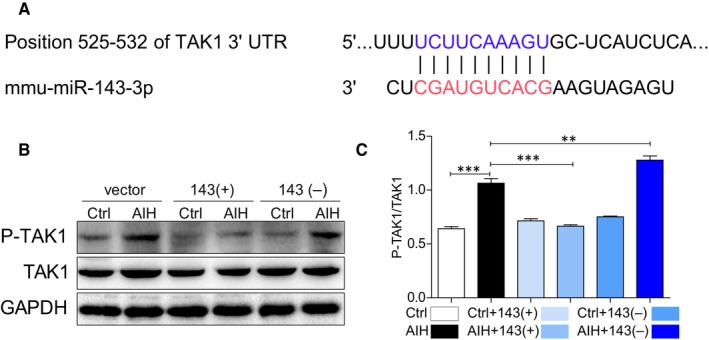
MicroRNA‐143 attenuates liver injury by targeting the TAK1 in vivo. A, The putative MIR143‐binding site in the 3' UTR of the uPAR‐PLAUR gene is predicted by TargetScan (http://www.targetscan.org/vert_71/), located at 525‐532 bp, respectively. B, Representative Western blot analysis of p‐TAK1 and TAK1 in liver tissues. GAPDH used as loading control. C, The right panel shows densitometric analysis. (*P < .05, **P < .01, ***P < .001, ns = no significance [n = 7‐9])

As shown in Figure 4A, TAK1 has been found to have a conserved miRNA‐143 binding site within its 3′‐untranslated region (UTR) in most species. Therefore, TAK1 was identified as the putative target to be regulated by miRNA‐143. Intriguingly, the protein level of TAK1 did not change between control and AIH no matter of any treatments but the phosphorylation level of TAK1 was significantly increased in liver tissues of AIH mice except mice treated with miRNA‐143 up‐regulation. More interesting, down‐regulation of miRNA‐143 aggravated the level of phospho‐TAK1 in AIH mice (Figure 4B and 4C). Therefore, it suggests that miRNA‐143 attenuates inflammation and fibrosis in AIH through regulation of TAK1 activation.

### 3.5 Result 5: MicroRNA‐143 mediates LPS‐induced inflammation in AML12 cells

It is well defined that LPS induces pro‐inflammatory cytokine expression through NF‐κB signalling pathway. To further investigate the mechanism of miRNA‐143 in regulation of inflammation and fibrosis, mouse hepatocyte cell line AML12 was employed to investigate LPS‐induced inflammation in cells.

**Figure 5:**
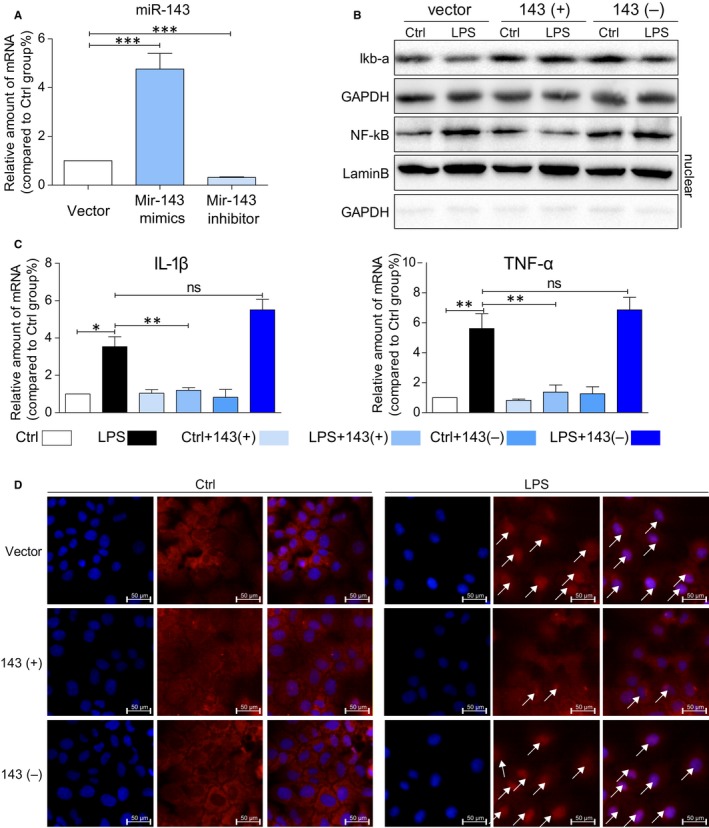
MicroRNA‐143 mediates LPS‐induced inflammation in AML12 cells. A, Mir143 up‐regulation and down‐regulation in AML12 cells. Cells were transfected with Mir143 mimic, Mir143 inhibitor or vector for 6h. mRNA levels of MicroRNA‐143 in the AML12 cells. B, Immunoblot analysis of iκB‐a and NF‐κB (nuclear) following miRNA‐143 up‐regulation and down‐regulation. GAPDH and lamin B used as loading control. C, mRNA levels of IL‐1β and TNF‐a on AML12 cells in each group. D, The AML12 cells in each group were stained with antibodies against p‐65 (shown in red) and counterstained with DAPI (shown in blue) (white arrow points the positive area). Cells were treated as in (B). (*P < .05, **P < .01, ***P < .001, ns = no significance, densitometric analysis of three independent experiments)

As shown in Figure 5A, transfection of miRNA‐143 into AML12 cells shows significant increase in miRNA‐143 in cells compared with transfections of vector and miRNA‐143 inhibitor. Moreover, LPS treatment also significantly stimulates the production of inflammatory cytokines such as IL‐1β and TNF‐a in cells transfected with vector and miRNA‐143 inhibitor while cells transfected with miRNA‐143, and there is no increase in IL‐1b and TNF‐a expressions (Figure 5C). Furthermore, LPS decreases iκB‐a protein and increases NF‐κB protein in the cells treated with LPS. However, miRNA‐143 treatment attenuates LPS‐induced alternations of iκB‐a and NF‐κB levels in AML12 cells (Figure 5B). Consistently, in Figure 5D, immunostaining data directly showed less positive staining of NF‐κB in the nucleus compared miRNA‐143 mimics pre‐treatment group to LPS and LPS with miRNA‐143 inhibitor. The results suggest that up‐regulation of miR‐143 can directly reduce LPS‐induced inflammatory responses in AML12 cells by mitigating the NF‐κB mediated inflammatory response.

### 3.6 Result 6: MicroRNA‐143 mediates LPS‐induced fibrosis in AML12 cells

**Figure 6:**
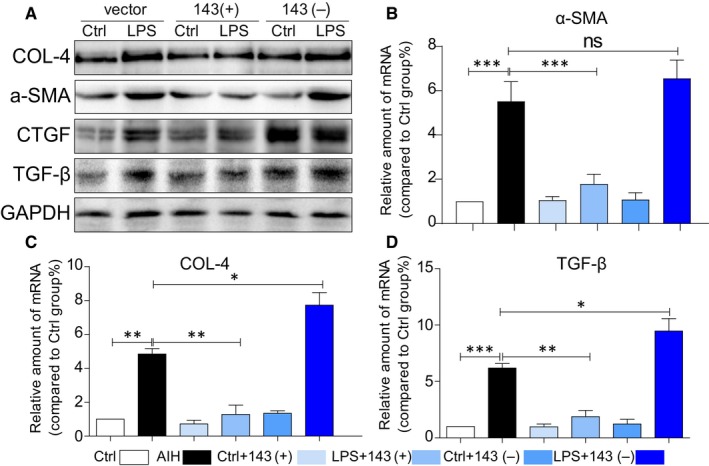
MicroRNA‐143 mediates LPS‐induced fibrosis in AML12 cells. A, Representative Western blot analysis of Col‐4, a‐SMA, CTGF and TGF‐β on AML12 cells. GAPDH used as loading control. B‐D, mRNA levels of a‐SMA (B), Col‐4 (C) and TGF‐ β (D) on AML12 cells. (*P < .05, **P < .01, ***P < .001, ns = no significance, densitometric analysis of three independent experiments)

We next determined the effect of miRNA‐143 on cellular matrix deposition in cultured AML12 cells. The effects of miRNA‐143 on production of collagen (COL‐IV), fibrotic cytokine (TGF‐β) and α‐SMA were also investigated in AML12 cells. When AML12 cells were transfected with vector, miRNA‐143 mimics and miRNA‐143 inhibitor, respectively, for 6 hours and then stimulated with LPS (0.5 μg/mL) for 24 h, the biomarkers of cell fibrosis were assessed by immunoblotting after incubating with LPS. There were significant increases in collagen IV, α‐SMA and TGF‐β (Figure 6A) after being co‐cultured with LPS and pre‐treated with miRNA‐143 mimics attenuated LPS‐induced cell fibrosis, while miRNA‐143 did not. Similar results are also shown in transcription levels (Figure 6B‐D). These findings indicated that miRNA‐143 can regulate expression of collagen, fibrotic cytokine (TGF‐β) and α‐SMA in hepatocytes.

### 3.7 Result 7: MicroRNA‐143 attenuates liver injury by inhibiting the phosphorylation TAK1 in vitro

**Figure 7:**
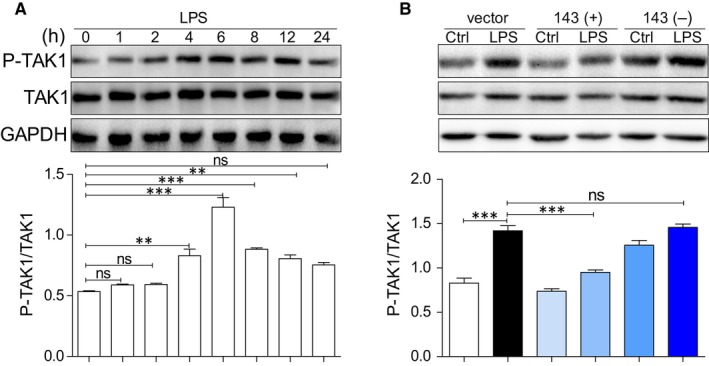
MicroRNA‐143 attenuates liver injury by inhibiting the phosphorylation TAK1 in vitro. A, TAK1 is rapidly phosphorylated by LPS. Immunoblot analysis of p‐TAK1 levels in AML12 cells exposed to LPS (0.5μg/ml) for different time periods (B) Representative Western blot analysis of p‐TAK1, TAK1 on AML12 cells. GAPDH used as loading control. (*P < .05, **P < .01, ***P < .001 ns = no significance, densitometric analysis of three independent experiments)

Whether miRNA‐143 can regulate phosphorylation of TAK1 in hepatocytes was shown in Figure 7. When AML12 cells were incubated with LPS (0.5μg/ml) for different time period, there is no significant increase in TAK1 protein level. However, significant increase in phosphorylated TAK1 was observed with maximum peak level at 6 hours after LPS treatment (Figure 7A). Therefore, cells treated with LPS at 6 hours were used to examine whether miRNA‐143 can regulate phosphorylation of TAK1. AS shown in Figure 7B, pre‐transfection with miRNA‐143 mimics prevents LPS‐induced TAK1 phosphorylation in AML12 cells and pre‐transfection with miRNA‐143 inhibitor escalated the level of phospho‐TAK1.

## 4 DISCUSSION

Autoimmune hepatitis is a rare clinical syndrome of immune‐mediated disorder of hepatocytes, typically with the development of auto‐antibodies,^22^ and if untreated, liver damage could lead to progressive injury leading to cirrhosis, hepatic failure or even death. Currently, the treatment of AIH mainly comprises corticosteroids in combination with azathioprine, and however, 38%‐93% of patients with this treatment achieve remission,^23^, ^24^ and up to 90% patients relapse after stop of therapy.^25^, ^26^ Moreover, 30%‐50% patients still develop cirrhosis despite treatment.^27^ Facing the high rate of relapse and serve side‐effects of conventional therapy, AIH remains a diagnostic and therapeutic challenge.

In recent years, several new therapeutic strategies have been emerged. For example, Chen et al shown that the mesenchymal stem cells (BMSCs) derived miR‐223‐containing exosomes contribute to liver protection in experimental autoimmune hepatitis,^28^ and Wu et al found that sodium butyrate ameliorates autoimmune hepatitis through regulation of intestinal tight junction and toll‐like receptor signalling pathway.^29^ In this study, we aimed to determine whether miRNA‐143 provides protection against AIH‐mediated inflammation and fibrosis in liver. With murine AIH model induced by liver antigen‐S100, we showed that up‐regulation miRNA‐143 mitigated liver injury during the AIH, while down‐regulation of miRNA‐143 worsen liver injury. The serum levels of ALT and AST were significantly elevated in S100‐induced AIH mice; however, they were significantly reversed in those mice treated with miRNA‐143 (+). Moreover, treatment of miRNA‐143 (−) did not reverse ALT and AST values in S100‐induced AIH mice. In addition, inflammation in the liver, which is characterized by high levels of TNF‐a in serum, down‐regulation of iκB‐α and high nuclear translocation of NF‐κB, was significantly relieved with miRNA‐143(+) transfection. Furthermore, the same effects of miRNA‐143 were demonstrated in AML12 cells challenged by classical inflammatory stimulator—LPS. The role of miRNA143 has been investigated in miRNA‐143/145‐/‐ mice, where it reported that several genes such as TGF‐β adaptor protein and disabled‐2 (DAB2) as well as the function of hematopoietic stem cells (HSC) were defected.^30^ Moreover, miRNA‐143 has been shown to regulate the NF‐κB and p38 MAPK pathways in CRC cells.^31^ Furthermore, miR‐143 level was decreased in patients with liver diseases especially liver fibrosis. In addition, the global miRNA profiling study revealed that miRNA‐143 and miRNA‐145 were differentially up‐regulated in rheumatoid arthritis fibroblast‐like synoviocytes (RA‐FLSs) than in osteoarthritis FLSs (OA‐FLSs).^32^ Therefore, our findings not only consistent with previous reports, but also complement the role of miRNA‐143 in protection of liver function in autoimmune hepatitis.

Since the most important outcome of autoimmune hepatitis are fibrosis and cirrhosis, severe complications of cirrhosis are upper gastrointestinal bleeding, hepatic encephalopathy,^33^ secondary infection,^34^ hypersplenism,^35^ ascites^36^ and cancer.^37^ Inflammatory and fibrogenic mediators produced by apoptotic hepatocytes and Kupffer cells in autoimmune hepatitis can promote the activation of hepatic stellate cells (HSCs), which further augment liver fibrosis by producing excessive extracellular matrix (ECM).^38^ Alteration of miRNAs target genes has been shown to involve in hepatic inflammatory, energy metabolism, cell regeneration and fibrogenic process.^39^ The miRNA‐143 has been documented to regulate the expression of matrix proteins and some signalling pathways. For example, miR‐143 was significantly reduced in post‐traumatic hypertrophic scar tissues and fibroblasts (HSFs). Moreover, treatment of HSFs with miR‐143 inhibited the protein expressions of collagen I (Col I), collagen III (Col III) and α‐smooth muscle actin (α‐SMA).^8^ This is consistent with the current finding in this study where miRNA‐143 regulates fibrosis in AIH mice liver. In the liver of AIH mice, the expression of collagen IV, a‐SMA, TGF‐β and CTGF in liver as well as CHI3L1 level in scrum was higher than those in healthy mice. With infection of AAV virus carrying miRNA‐143, levels of fibrotic parameters in the liver and serum were significantly reduced and the liver appears normal in size and shape. In addition, our results are also consistent with miRNA‐143‐regulated extracellular matrix gene expression in smooth muscle cells as well as miRNA‐143 suppresses TGF‐β‐dependent extracellular matrix accumulation and fibrosis in smooth muscle cells.^40^

In order to understand potential mechanism of miRNA‐143 in alleviation of liver injury in AIH, we explored the microRNA database (miRBase at http://www.mirbase.org), which is the high confidence microRNA data set^41^ to identify potential target of the miRNA‐143. With the TargetScan miRNA target prediction tool,^42^ we have identified that TAK1 may be one of the key miRNA‐143 targets. It is known that TAK1 can be activated by numerous receptors that mediate inflammatory and fibrosis signal transduction pathways in response to ligands such as transforming growth factor‐β (TGF‐β), toll‐like receptor (TLR) ligands, tumour necrosis factor α (TNFα) and interleukin‐1β (IL‐1β).^16^ Attenuating activation of TAK1 showed inhibition of scar formation.^43^ Moreover, in non‐alcoholic fatty liver disease (NAFLD), dual‐specificity phosphatase 26 (Dusp26) can bind to TAK1 to prevent phosphorylation of TAK1 to alleviate hepatic steatosis and metabolic disturbance.^44^ Consistently, our results showed there were a significant increase in TAK1 phosphorylation and a decrease in iκB‐α in the liver of murine AIH model. Moreover, treatment of AAV‐miRNA‐143 significantly reduced TAK1 phosphorylation and elevated iκB‐α in the liver of AIH mice. However, AAV‐miRNA‐143‐scramble sequence did not have any effects on either phosphor‐TAK1 or iκB‐α. Furthermore, the results from LPS challenged AML12 cells showed similar findings obtained from AIH mice study. The findings from the current study suggest that TAK1 may mediate the development of AIH through its phosphorylation and activation of iκB‐α degradation, as well as miRNA‐143 may prevent TAK1‐medicated events in AIH mice. Therefore, the current study identified a novel target of miRNA‐143, regulation of which could contribute beneficial effects on AIH‐induced liver inflammation and fibrosis. However, AIH is a complicated disease and will not be able to be healed by one target of miRNA‐143, and miRNA‐143 should have multiple target genes.

In conclusion, our results provide the evidence that overexpression of miRNA‐143 attenuated inflammation and fibrosis of autoimmune‐induced liver injury through regulation of phosphorylation TAK1 in both the liver of AIH mice induced by hepatic antigen S100 and LPS‐induced hepatocyte injury. However, its clinical application requires further research. The distribution of p‐TAK1 in the liver of patients with autoimmune hepatitis still needs to be explored. Moreover, the level of miRNA‐143 in liver and scrum of AIH patients also need to be examined. With the current evidence, TAK1 in the liver could be the potential target to develop a promising genetic therapeutic agent for AIH.

## CONFLICTS OF INTEREST

All the authors declare no competing financial interest.

## AUTHOR CONTRIBUTIONS

YC and LX designed the research study. HT performed the research. HT, DC, CC, TP, YX and TT analysed the data. QD, HL, JT, LS, ZL, XW and RW contributed essential reagents or tools. YC and HT wrote the paper and submitted the final versions.

## Data Availability

All data generated or analysed during this study are included in this published article [and its Appendix S1]. And all data used to support the findings of this study are available from the corresponding author upon request.
