# Supplementary material for: microRNA‐143‐3p attenuated development of hepatic fibrosis in autoimmune hepatitis through regulation of TAK1 phosphorylation
Source: J Cell Mol Med. 2019 Dec 6;24(2):1256–67. doi: 10.1111/jcmm.14750 (PMC6991639; doi:10.1111/jcmm.14750)
Supplement: Supplementary file 1 [file JCMM-24-1256-s001.doc]

***Supporting Information***

**microRNA-143-3p attenuated development of hepatic fibrosis in autoimmune hepatitis through regulation of TAK1 phosphorylation**

Hanxiao Tu^1,#^, Dazhi Chen^2,#^, Chao Cai^1^, Qianjing Du^1^, Hongwei Lin^1^, Tongtong Pan^1^, Lina Sheng^1,3^ , Yuedong Xu^1^, Teng Teng^1^, Jingjing Tu^1^, Zhuo Lin^1^, Xiaodong Wang^1^, Rui Wang^1^, Lanman Xu^1,4,5^*, Yongping Chen^1^,*

*^1^ Department of Infectious Diseases, The First Affiliated Hospital of Wenzhou Medical University, Hepatology Institute of Wenzhou Medical University, Wenzhou Key Laboratory of Hepatology, Wenzhou 325000, Zhejiang, China*

*2 Department of Gastroenterology, The First Hospital of Peking University, BeiJing, 100034, China*

*3 Department of Infectious Diseases, The Affiliated Yiwu Central Hospital of Wenzhou Medical University, Yiwu, 322002, Zhejiang, China*

*4Department of Infectious Diseases and Liver Diseases, Ningbo Medical Center Lihuili Hospital, Ningbo 315040, Zhejiang, China*

*5 Department of Infectious Diseases and Liver Diseases, The Affiliated Lihuili Hospital of Ningbo University, Ningbo 315040, Zhejiang, China*

**Running Title:** microRNA-143-3p attenuates autoimmune hepatitis via TAK1.

**#, These authors contribute equally to this page**

***Corresponding authors.**

***Correspondence to:**

Prof. Yongping Chen, at the Department of Infectious Diseases, The First Affiliated Hospital of Wenzhou Medical University, Wenzhou Key Laboratory of Hepatology, Hepatology Institute of Wenzhou Medical University, Wenzhou 325025, China.; Telephone: +8613505777281; E-mail: did@wzhospital.cn (Prof. Y.p. Chen).

Prof. Lanman Xu, at the Department of Infectious Diseases and Liver Diseases, The Affiliated Lihuili Hospital of Ningbo University, Ningbo 315040, Zhejiang, China.; Telephone: +8613587646315; E-mail: xulanman@163.com (Prof. L.m. Xu).

**Table S1 The primer sequences of genes in real-time qPCR assay**

| **Gene** | **Species** | **Forward** | **Reverse** |
| --- | --- | --- | --- |
| TNF-a | Mouse | TGATCCGCGACGTGGAA | ACCGCCTGGAGTTCTGGAA |
| IL-1β | Mouse | ACTCCTTAGTCCTCGGCCA | CCATCAGAGGCAAGGAGGAA |
| TGF-β | Mouse | TGACGTCACTGGAGTTGTACGG | GGTTCATGTCATGGATGGTGC |
| Collagen VI | Mouse | GCTCCACCACTCAAAGGTGTT | GGCACAGTCGAGTCTTCCA |
| α-SMA | Mouse | GTCCCAGACATCAGGGAGTAA | TCGGATACTTCAGCGTCAGGA |
| β-actin | Mouse | CCGTGAAAAGATGACCCAGA | TACGACCAGAGGCATACAG |
| miR-143-3P | Mouse | CGGGCTGAGATGAAGCACT | CAGCCACAAAAGAGCACAAT |
| miR-143-5P | Mouse | CGCCGGGTGCAGTGCTGCA | CAGCCACAAAAGAGCACAAT |
| U6 | Mouse | GCTTCGGCAGCACATATACTAAAAT | CGCTTCACGAATTTGCGTGTCAT |


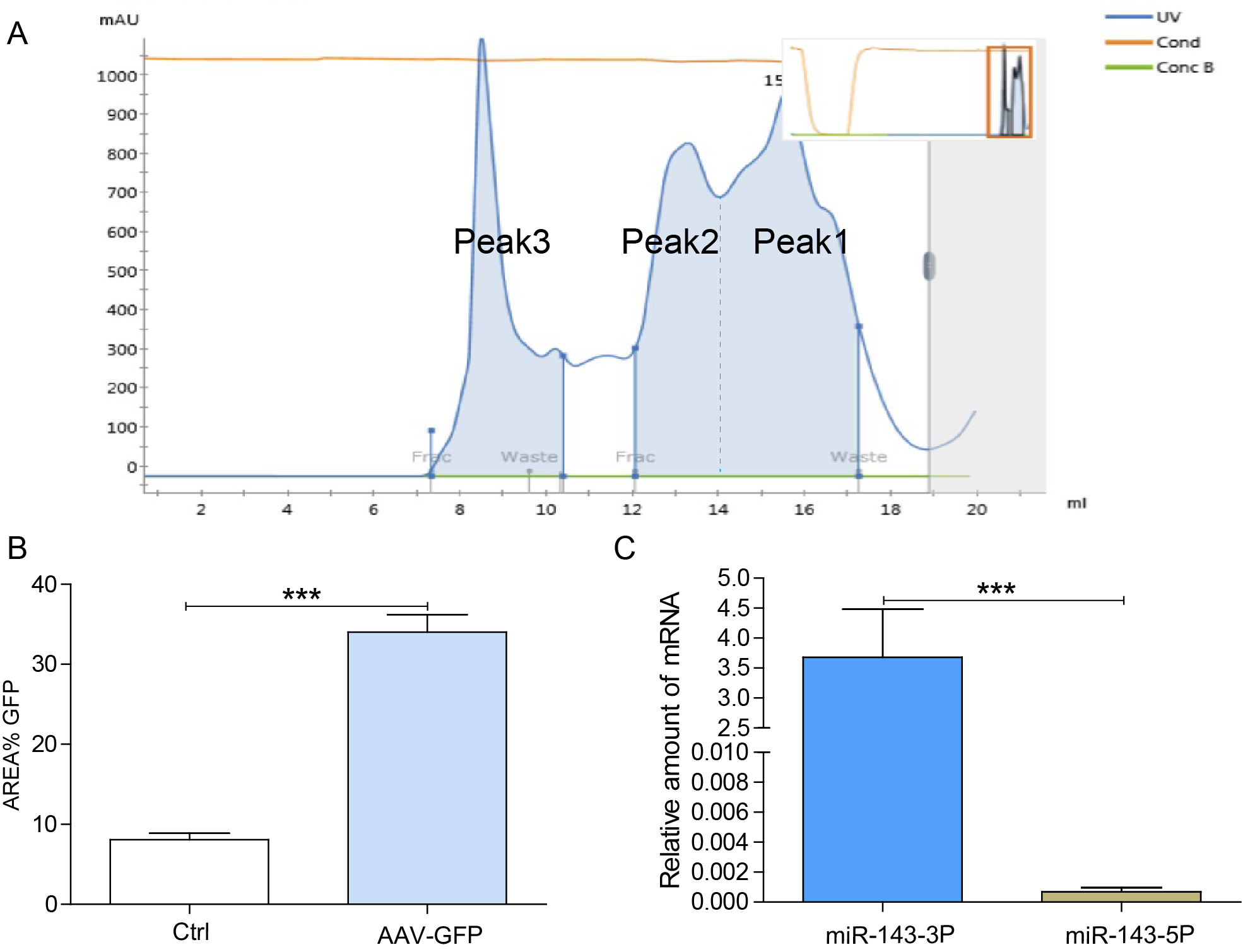


**Supplementary Figure S1:** (A) The protein peak map of mice liver homogenate separated by the AKTA. The peak 1 (small molecular fraction) components was chosen to induced AIH in mice;(B) Densitometric quantification of data shown in Figure 1C; (C) mRNA levels of miR-143-3P in the Ctrl-mice liver tissues (*P < 0.05, **P < 0.01, ***P < 0.001 compared to vector-Ctrl, ns = non-significance [n=7-9]);


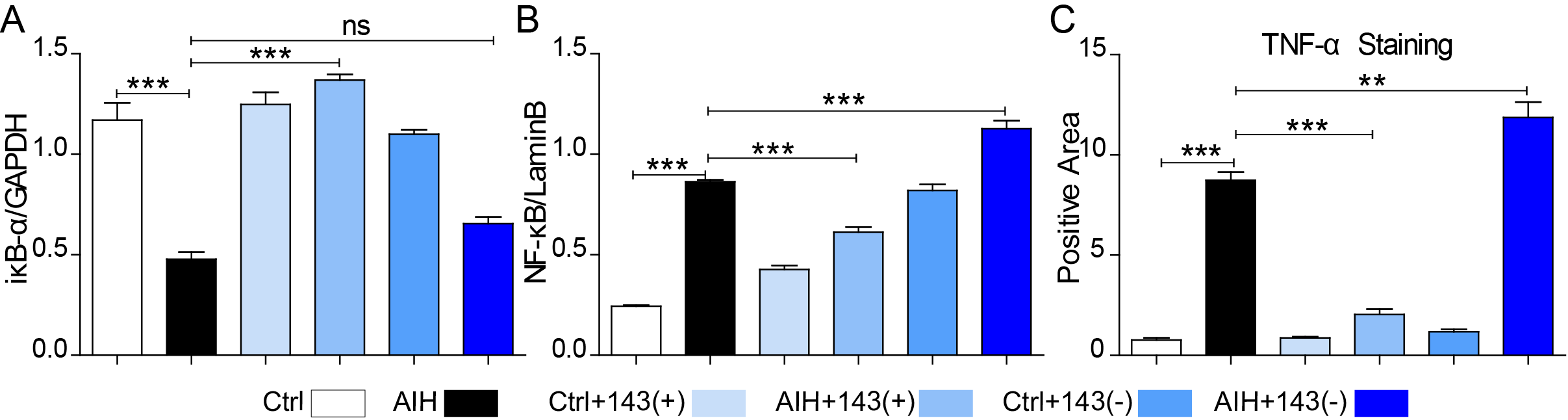


**Supplementary Figure S2:** (A-B) Densitometric quantification of data shown in Figure 2C ;(C) Densitometric quantification of data shown in Figure 2E. (*P < 0.05, **P < 0.01, ***P < 0.001 compared to vector-Ctrl, ns = non-significance [n=7-9])


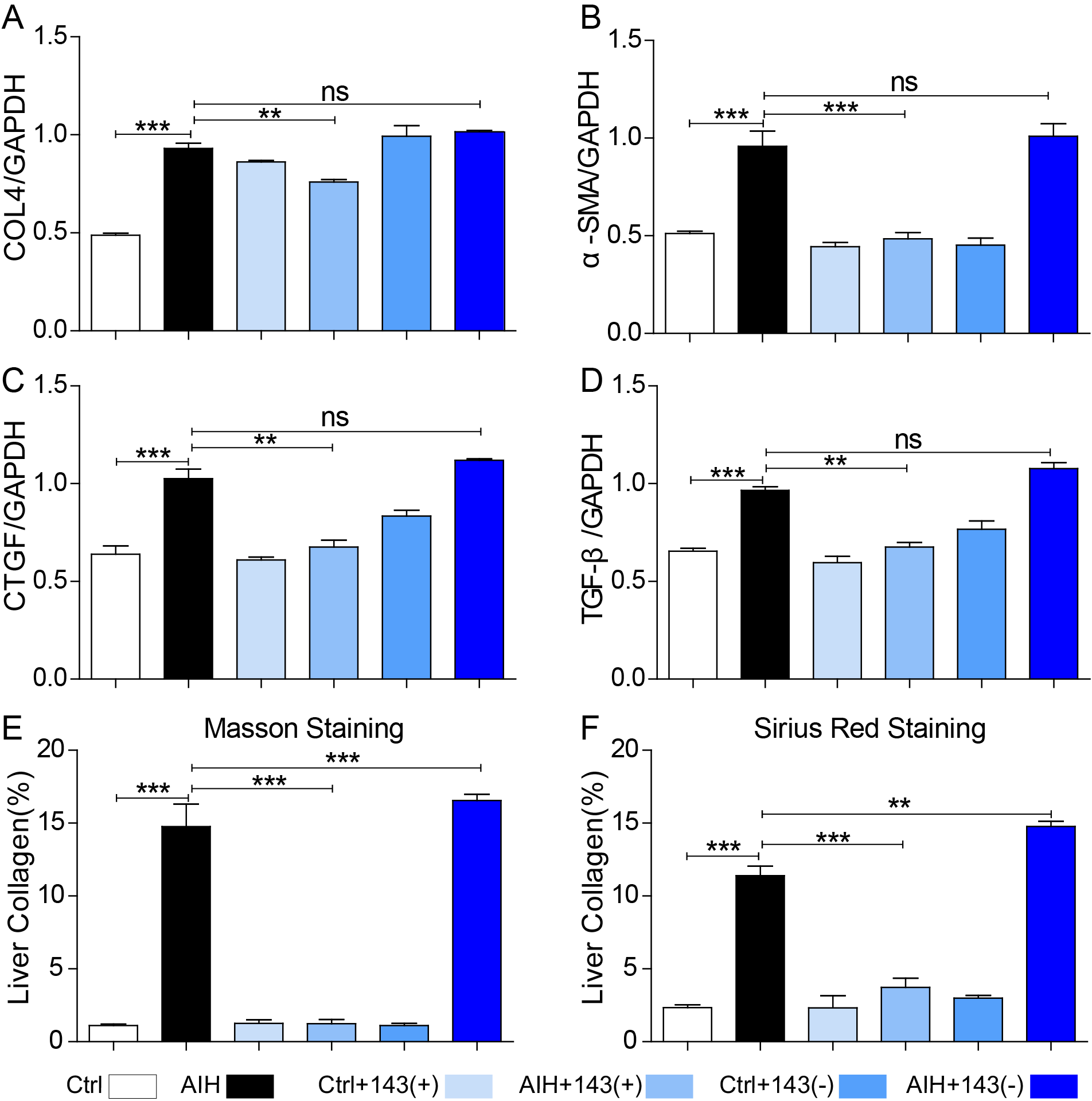


**Supplementary Figure S3:** (A-D) Densitometric quantification of data shown in Figure 3B ;(E-F) Quantification of interstitial fibrotic areas (%) from Masson’s Trichome staining (E) and Sirius red-stained heart sections (F) in Figure 3E-F. (*P < 0.05, **P < 0.01, ***P < 0.001 compared to vector-Ctrl, ns = non-significance [n=7-9])


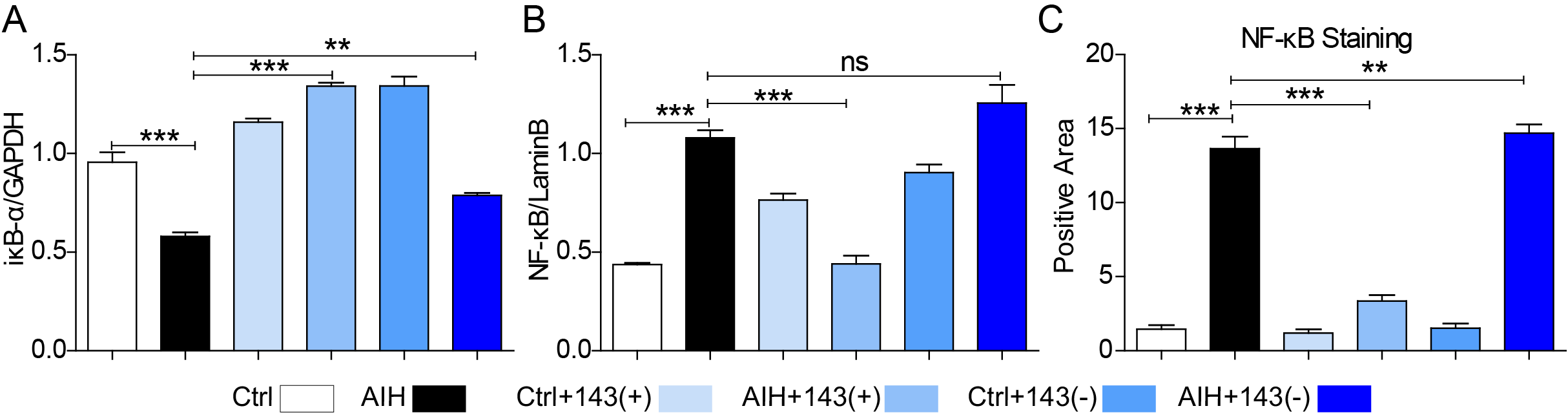


**Supplementary Figure S4:** (A-B) Densitometric quantification of data shown in Figure 5B ;(C) Quantification of positive immunoreactivity area (%) from NF-κB staining in Figure 5D. (*P < 0.05, **P < 0.01, ***P < 0.001 compared to vector-Ctrl, ns = non-significance [n=3])


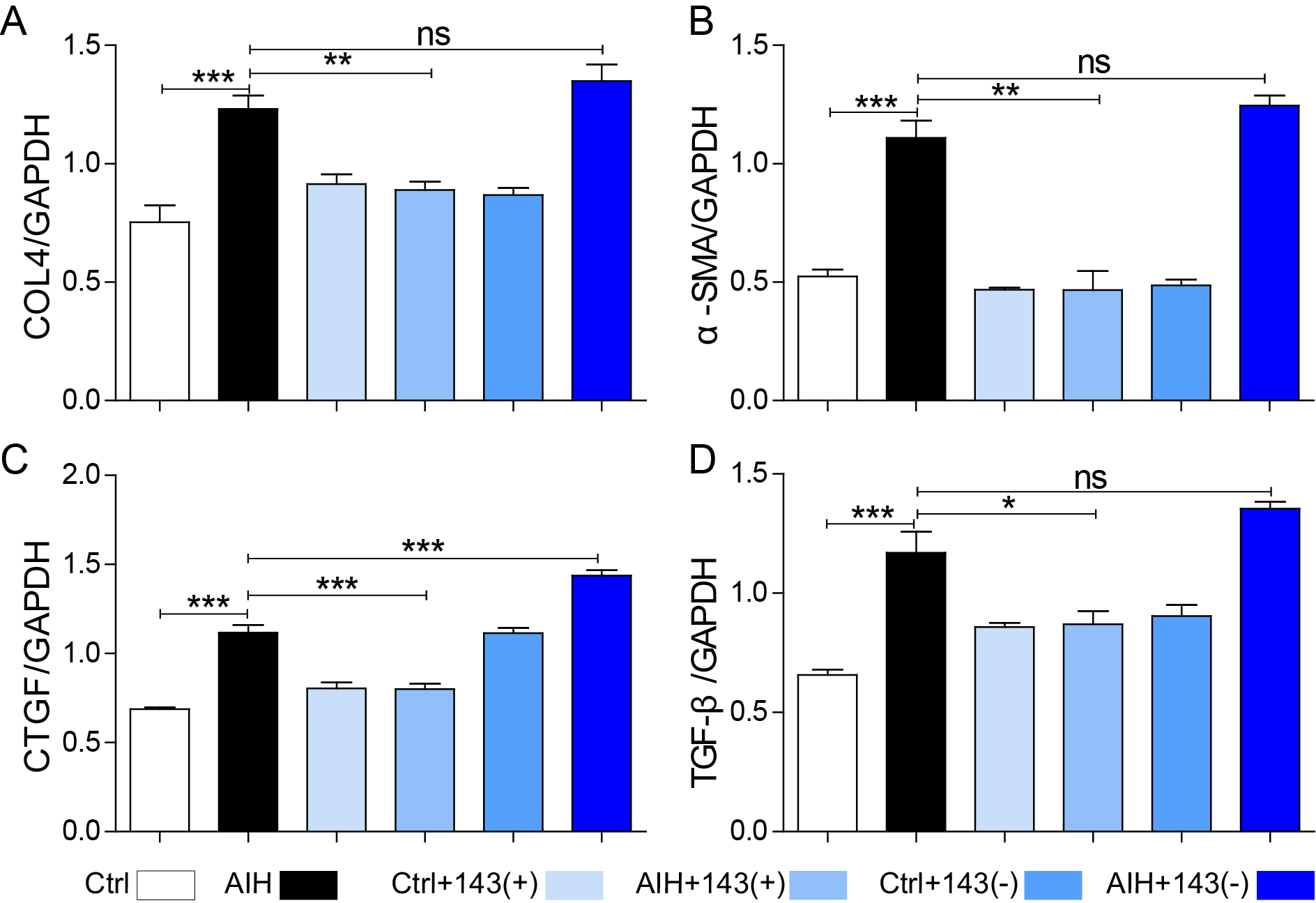


**Supplementary Figure S5.** (A-D) Densitometric quantification of data shown in Figure 6A; (*P < 0.05, **P < 0.01, ***P < 0.001 compared to vector-Ctrl, ns = non-significance [n=3])
